# Dr. Buchan on Symptomatology

**Published:** 1825-04-01

**Authors:** 


*Symptomatology, or the Art of detecting Disease, a Lecture occasionally read to the Pupils at the Westminster Hospital, and published according to their Request. To which are added Tables of Symptoms.* By Alex. P. Buchan, M.D. F.L.S. late Senior Physician to that Institution, and Member of the Royal College of Physicians, London. 12mo. pp. 190. London, 1824.

Symptomatology has not hitherto taken its due station in the course of medical studies,—at least, in this country. It is but very lately, indeed, that we have been put into possession of the *first* distinct treatise on this subject, if we except the very inadequate and imperfect performance of Dr. Berkenhout, published in 1784; we, of course, allude to the Essay of Dr. Marshall Hall, of which we gave a review in our number for March last. And, considering that there is certainly no dearth amongst the fruits of the medical press, we cannot but remark this fact, as perfectly extraordinary and unaccountable.

It will be our object, in the course of the subsequent observations, to draw the attention of our readers to the subject of symptomatology, by pointing out to them the all-important and essential nature of this department of medical science, and the still inadequate mode and degree in which it has been investigated and developed. We undertake this task with the more satisfaction, because we have the confident hope that it will induce some individuals to take up the enquiry; and, especially, that it will, as we trust, stimulate that physician who has already laboured in this fruitful field, to continue his exertions, by observing that they are, at length, obtaining the notice they merit with the profession at large.* He will perceive that we have not hesitated to borrow some ideas from his little volume, in pursuing our present strictures on the subject of symptomatology.

Our first remark, with the view of establishing the importance of this study, is, that it is, in fact, the only one which *can avail us in the sick-room or ward.* It is the only clew which can lead us out of the labyrinth of our doubts or per-

* We understand that the Essay on the Symptoms and History of Diseases is again out of print, and that a third edition is in the press.

plexities respecting the nature and identity of the disease, and respecting the effects of the remedies which have been administered. Whatever our views may be, they are only to be established or corrected in practice, by the symptomatology. The diagnosis and the prognosis, the indications and the effects of remedies, are equally learnt from the same source.

Our second observation is, that ALL our other knowlege is made *practically useful* ONLY *through the medium* of symptomatology. Of what *use* is the most minute and perfect knowledge of morbid anatomy, unless we have the means of ascertaining its existence in the living body? The facts in morbid anatomy are valuable, as all facts in science must be acknowledged to be; but they are *practically* useful only in the degree in which we are enabled to establish their existence in our patients, and this can only be done through the medium of symptomatology.

What then can have been the reason, that this most important of medical studies has been so long neglected by our practical writers, and that there should be but two or three works on the subject in our language? It is, we think, on account of its great difficulty. There is no study which requires so much cautious observation, so much patient reserve, so much repetition, in coming to our conclusions, as that of symptomatology. If a hasty observer were to give us a system of symptomatology, it might be diffuse, but it would want all that is valuable in this department of medicine—namely, *great accuracy*. Many symptoms occur in the course of a disease, which are not essential to it, or at all characteristic of it, but perhaps the result of some complication, or the effect of some remedy. Cautious and repeated observation alone can ascertain these facts, can distinguish between those symptoms which are essential and those which are accessory. If these be blended in a treatise on symptomatology, all is confusion. If there be, then, one qualification for the writing of such a work more absolutely necessary than another, it is *reserve*. Thus the beginning of a disease is often characterised by a different series of symptoms from its middle and later stages; and especially towards the conclusion of a disease, many changes take place in the morbid anatomy, and in the state of the vital powers, all which must be accurately observed and re-observed and associated with their appropriate symptoms, before any sound advancement can be said to be made in the study of symptomatology.

These observations are completely confirmed on reading some of the works written on this subject. After almost every paragraph, we are apt to stop and say "this is vague," "this is doubtful." And this leads us further to remark, that another cause of the neglect of symptomatology by our practical writers has probably been the manifest dissatisfaction experienced on reading those treatises which have been written on this subject. This defect is not, however, we venture to say, inherent in the subject itself—but the result of a too hasty and unripened observation. In this study, especially, we all want our wings clipping, and must be content to proceed very slowly; we shall then, however, be enabled to note useful facts in symptomatology, and to form useful, because cautious, associations of the symptoms as the sign, and of the morbid state as the thing signified. And whatever difficulties beset us in our attempts to *write* on symptomatology, precisely similar difficulties attend the application of our knowledge of morbid anatomy to the practical purposes of our art. It is useless to write on symptoms, except as *signs* of diseases, and morbid anatomy is equally useless, except it be ascertainable in the living patient by such *signs*. In both departments of medical science it is the accurate association of the symptoms and of the disease, which constitutes at once the great difficulty, and the practical utility of our investigations.

Attended and surrounded as it is by all these difficulties, symptomatology is thus, then, the only part of our knowledge which is available in actual practice. This fact, which is undeniable, *must* arrest our attention. And we trust it will not fail in procuring from our readers all that consideration which is due to the subject.

It is said to have been a favourite saying with the late Dr. Cullen, that there are, in physic, more *false facts* than *false theories*. The observations which we have already made, will, we think, suggest the probable foundation for this remark, admitting it to be true. But the remark itself should lead us to inquire into the causes of this fallacy respecting our conclusions and supposed facts in medicine, and into the probable mode of obviating it in future. If our facts be "false," it must either be from there being but a *defective* evidence for them, or from the evidence we have being imperfectly brought to bear upon them. In order to determine these points, it will be necessary to recur to the sources of our evidence for facts in medicine, and to those mental processes by which this evidence is seized and elaborated into use.

It is obvious, in the first place, that all our knowledge in regard to disease, is obtained through the medium of the *senses*, especially those of sight, hearing, and touch, though the sense of smell is by no means useless to the attentive and practical physician. It does not appear to be necessary to insist upon the great importance of an assiduous cultivation of the powers of these inlets to our knowledge. But it may not be entirely useless to urge upon our junior readers, for whom these remarks are chiefly intended, the great advantage of an *early* cultivation of the powers of our senses, and especially of those processes of the understanding habitually and almost unconsciously attendant upon our perceptions. Dr Buchan observes

"It is now generally admitted, that all our ideas, all those at least concerning external nature, are conveyed to the perceptive principle through the medium of the senses. But the several senses do not transmit their impressions to the mind in the same manner. The senses more immediately subservient to the support of individual life, touch, smell, and taste, are excited by immediate contact of the substances, with the qualities of which they make us acquainted. But the eye is affected through the medium of the rays of light, and the ear through that of the vibrations of the air. The information derived from the former senses may be termed immediate, from the latter mediate. Of the former class of sensations, that chiefly employed in the investigation of disease, is the touch, from which however the surgeon derives more knowledge than the physician.

"To enable us to detect the nature of the various diseases to which the complicated structure of the human body renders it liable, it is our duty to cultivate the senses, through which we derive a knowledge of their nature; which, let it be observed, are, like all the other faculties of man, capable of being improved. We should endeavour to acquire the tactus eruditus, the visus eruditus, and now that it is found practicable to detect affections of the lungs by sounds produced by striking upon the thorax, also the auditus eruditus." 16.

That faculty which has generally been termed *tact*, is by no means mere pretending or idea. It is the result of an assiduous cultivation of the senses, and of the powers of perception and judgment; for it is to be observed, that these processes ever follow the exercise of the sense. We cannot illustrate this proposition more clearly and forcibly than by an allusion to the act of feeling the *pulse*. How much the physician learns from this act, by a process of reasoning of which he is scarcely conscious, and the analysis of which would certainly cost him much trouble, but would amply repay his curiosity! He takes the peculiar modification of the pulse, in connexion with *all* the circumstances of the patient, in an inconceivably short space of time;—the age, the disease, the remedies which have been administered, each modify the pulse, and are each taken rapidly into the account, in his estimation of its value and its indications; if the pulse be more frequent than it was anticipated it would be, every cause that might add to this frequency is made to pass in review through the mind, and the effects of corporal exertion, of mental emotion, of an error in diet, or of an augmentation of the malady, are estimated almost instantaneously. Similar remarks apply to all the other objects of our perceptions. But we have already stated sufficient to illustrate the *principle*, which is all that our space will enable us to do. We have only once more to press upon our young readers the importance of cultivating the faculties of perception —the spirit of observation—and that inestimable habit, at once, of perception and of judgment, involved in our idea of *tact*.

The faculty of which we have just spoken relates to our perception and judgment of each individual object of sense, in the persons of our patients. There is another faculty of equal moment, and even perhaps of a higher order, because still more complicated and refined, which may be termed the *coup-d'œil* of the physician. In tactics, such a faculty has been termed the *military eye;* and it is said by Livy, to have been possessed in a remarkable degree of perfection by Philopœmen, of whose studies, and of the modes by which he acquired this quickness of tactic perception, he gives an accurate and interesting account. In this account, *three* things may in particular be observed,—that the faculty was acquired, "*curis, cogitationibusque*,"—and "*ab ineunte ætate*," and that in forming his judgment, "*contemplatus est ab omni parte loci natura*." These are the very points for acquiring the *medical* as well as the military coup-d'œil. It must result from much observation and thought; it must be cultivated in our early years; and it must be formed on *all* the circumstances of the patient and of the case. Dr. Buchan's remarks on this subject are highly interesting.

"But the capacity of discerning the nature of disease varies widely in different individuals. 'Quicquid recipitur, recipitur modo recipientis.' I have known instances of medical men, who even after long-continued experience could never clearly ascertain the nature and distinction of diseases, ncr even put questions pertinent to their investigation; while others, as was said of the celebrated Fothergill, appear to possess an almost intuitive perception of the state of their patients. The possession or the absence of this faculty constitutes, in fact, the main difference between one medical practitioner and another. What Lord Bacon says of love, seems applicable to disease: 'Love,' says he, 'is not manifested by staring in the face, but is communicated by slight glances, and sudden quick sparkles of the eye." 17.

"We should be careful not to trust too much to the replies of patients or their friends to our verbal inquiries in the investigation of disease, but in preference attempt to interrogate nature herself. Doubtless there exists a pathological physiognomy well worth the attentive study of the industrious practitioner. Every internal disease of a serious nature imprints upon the countenance of the patient a certain cast, or air, from which the attentive physician may derive an important diagnostic.

"It is related of the celebrated Dr. Stoll of Vienna, that he could distinguish the trade of every artisan, who applied to him for advice, by the peculiarity of his manner. I am myself acquainted with an individual, who possesses a considerable share of this peculiar discriminative faculty. Let us reflect, that the veterinary practitioner, and those persons, who make infantile diseases the chief object of their attention, have no other means of guiding their judgment than attentive observation. The possession of this discriminative faculty in perfection constitutes what has been termed the scientific tact, and forms, perhaps, the ultimate perfection of medical talent." 21.

The object of this faculty is the *general* aspect of the patient, and combinations of the symptoms. The processes of the perception of these various objects, of the reasoning founded upon that perception, and of the judgment which flows from this reasoning are so rapid, that the knowledge thus obtained, appears to be almost intuitive. But it is, in point of fact, the result of long habit of observing all the circumstances of the patient, first *seriatim*, and then in their various *combinations.* We must not enlarge upon this subject, but before we do quit it, we must be allowed to point out, that a great point is to *feel* the force of a consistency or of a discrepancy in the combinations to which we have last alluded. Acute pain in pleurisy, for example, prevent the patient from speaking in a loud and irascible tone of voice. The manner of the patient should then be consistent; and if it be not, we must either suspect that the pain is not so severe as it is represented to be, or not of an inflammatory character.

The cultivation of this talent must not, however, induce us to neglect the following judicious advice of Lord Bacon:

"Men use commonly to take a prospect of nature, as from a high turret; and to view her afar off; and are too much taken up with generalities. Whereas, if they would resolve to descend, and approach nearer to particulars, and more exactly and considerately look into things themselves, there might be made a more true and profitable discovery and comprehension. The remedy of which error is, to go nearer to the object. And, therefore, there is no doubt, but if physicians, letting go generalities for a while, and suspending their assent thereto, would make their approaches to nature, they would become more complete masters of their art, and better enabled to accommodate their means of cure to the various states and conditions of diseased actions." 56.

Besides the cultivation of *tact*, and of the *medical eye*, to which we have already alluded, there is a species of *mental discipline*, more purely so termed, to which we should all submit ourselves. We shall exemplify the object of this mental exercise very briefly. And we would first observe, that the evidence for facts in physic is certainly not so indubitable, but we may, or rather do, frequently form wrong notions of the nature of a case. We should, then, cultivate great candour of mind, and, ever alive to fresh appearances, be ready on all occasions to rectify our opinions, if ulterior appearances should seem to point to a case of a different nature. In the second place, we should be ever aware that internal disease may not only be in a state of progress, but may involve other organs than those first affected. We should be constantly on our guard duly to appreciate these changes in disease. This remark is especially exemplified in the cases of fever, inflammation, and tuberculous disease: the first may be originally simple, but may in its course receive a most serious modification by the addition of some local disease in the head, chest, or abdomen; inflammation may be, in its commencement, confined to one of the abdominal viscera, but it may afterwards involve all the organs covered by the peritoneum, and even affect those of the chest; and it is very frequently observed that tubercles, although the symptoms may attach originally to the abdomen, pervade not only that cavity, but that of the thorax too, as well as other parts of the body. Let us not take *too simple and contracted* a view of cases; but let our inquiries occupy an enlarged sphere. In the third place, let us, in contemplating the disease, ever duly appreciate the superadded influence of the various potent remedies which may be employed; let us, in combating inflammation, have a just regard to the strength of the patient, and not, whilst vanquishing one foe, raise up another to be opposed in its turn. Pietro Aretino used to compare medicine to a torrent, which not only carries away with it the stones from a field, but likewise a good part of the field itself.—Verbum sat sapienti.

Another part of mental discipline is ever to be guarded against implicitly receiving the answers of the patient himself to the questions we may put to him. Dr. Buchan judiciously observes,—

"A variety of circumstances render it often difficult to elicit from patients the truth, which we wish to discover. Internal sensations are always obscure, and therefore difficult to express, and the perception of them is frequently disturbed by the presence of unhealthy action. Disease, existing in one particular viscus, is frequently indicated by pain perceived, in a distant part of the body: affections of the liver, for example, by pain in the shoulder. In examining patients, we have moreover to contend with anxiety of mind, with unintentional misrepresentation, arising from the object of our queries not being understood. Clearness of intellect too is more or less obscured by the presence of disease, and thus common forms of expression do not always convey the same ideas to the patient and the practitioner. Even the friends of patients frequently mislead by what they consider as well-meant misrepresentations respecting the previous habits and conduct of the sick person." 19.

"Unnecessarily to multiply our questions is always distressing to a patient. Irritability of temper is generally the concomitant of disease, and I have often observed, that sick persons will frame such replies as you seem to expect, rather than be teased with a protracted examination. This is probably one reason why many persons resort to those, who pretend to discover the nature of diseases from the inspection of the urine alone, without asking any questions. Patients are moreover prone to consider too many questions as implying ignorance of the nature of their complaint. A lady once asked me in a peevish tone, how those persons, who cured horses and dogs, found out what was the matter with them. We should, therefore, carefully endeavour to make ourselves acquainted with every obvious indication of disease, that may tend to diminish the necessity of troubling our patients with a multitude of interrogatories." 80.

Having thus briefly sketched our view of the mental processes by means of which we are enabled to collect the evidence for facts in clinical medicine, we now proceed still more shortly to state the *sources* from which that evidence flows. These are, then, the symptomatology, the history, and the morbid anatomy. It need not be concealed, that the last of these departments of study is that which, in the present day, "in honore est." We have, lately, repeatedly delivered our opinions on this subject. The reader may consult p. 63 of the present volume. From those observations, and from the remarks contained in the beginning of this article, it will be readily collected, what we think on this still most important department of medical study. We would avoid the usual error of mankind, of passing from one extreme to another, from the extreme of overrating the importance of morbid anatomy, to that of denying its real value. The study of morbid anatomy has added greatly to our stock of knowledge, and has thus greatly benefitted the practice of physic; but, as we have already said, in our actual visits to our patients, it is *not* the morbid anatomy, but the symptomatology which is our only and constant guide. We believe we state the truth too, when we allege that the English have studied the morbid anatomy to the neglect of the symptoms, and that the French have done still worse, by investigating the pathology, to the neglect of what is the *end* of all—the remedy. We, however, in this Journal, will endeavour to place these matters in their real and true points of light;—letting even-handed Justice hold the balance, we shall give to each of these branches of study its real weight and importance.

On the *history* of diseases, far too little has been written. The slight sketch of this source of the evidence for facts in clinical medicine, in Dr. Hall's work is almost the only one, indeed the only one, to which we can refer. We trust, therefore, that he will do the subject still more ample justice, in the forth-coming edition of his useful Essay.

We have hurried over this subject, in order that we might again take up Dr. Buchan's little volume. We own we are rather astonished that, considering that Dr. Hall had so lately trod the same path, Dr. Buchan does not once mention his fellow-traveller. It was the least shew of courtesy that could be expected. Dr. Buchan's work consists of an introductory lecture from which we have already made some extracts, and tables of symptoms, "chiefly copied from the symptomatology of Berkenhout." That something far better, and far more useful and satisfactory, might have been written, or even compiled on this subject, we think we need not say. Still Dr. Buchan's work contains *some* good remarks; of these we shall make a selection, and with that conclude, for the present, our observations on symptomatology. We purpose, however, shortly to take up the subject again, to give some account of some foreign works upon it, especially those of M M. Landré-Beauvais and Double.

Dr. Buchan observes "the first question we put to a patient, is whether he feels *pain;* and the next. where the pain is seated." These are, at least, very important questions; and we are persuaded that an interesting essay might be written on the subject of pain alone; it would especially be important to give a *list* of the different kinds of pain—of those species of pain which portend disease—of those which occupy a seat distant from that of the disease, &c. We proceed to extract some observations on pain by our present author, as a specimen of his mode of treating his subject, without adding any comment, or being supposed to agree or disagree with him on the several points noticed by him.

"Pain in the front of the head generally indicates fulness of blood; in the occiput, or hind-head, exhaustion, or nervous debility, frequently consequent to fatigue." 25.

Fixed pain seated over one of the eyes, which has been termed clavus hystericus, indicates torpor or debility of the stomach, generally removable by warm, bitter eccoprotics. Sudden darting pains in the head, more especially if accompanied with perception of heat on the hand being applied to the forehead, dilated pupil, and strabismus, or squinting, in young persons, is indicative of the approach of hydrocephalus, a disease requiring the most energetic remedies."

"Pain of the small of the back is the precursor of fevers, particularly of the eruptive kind, and often indicates disease of the uterus. Acute pain of the bowels indicates inflammation, diarrhœa, dysentery, &c."

"Pain in the neighbourhood of the large joints indicates what is termed rheumatism; but if seated towards the middle of the long bones, especially if aggravated during the night, there is reason to suspect the existence of latent syphilis."

"Aching of the knee is symptomatic of disease of the hip-joint."

"Obtuse pain in the right hypochondrium, darting towards the scapula and top of the shoulder, is symptomatic of torpor or disease of the liver.

"A sense of pain and weight in the loins, accompanied with nausea, indicates nephritis, or inflammation of the kidney."

"A sense of pain and heaviness in the loins often precedes an attack of hemorrhoids, and the first appearance of the menses in females. Pain seated at the scrobiculus cordis indicates scirrhus of the stomach or liver: if very severe, and accompanied with costiveness, the presence of biliary calculi. Patients complain of this kind of pain as peculiarly severe."

"The recurrence of pain in a part formerly bitten by a rabid animal, even at a remote period, betokens the approach of that dreadful disease, hydrophobia." 31.

The following observations are made respecting the *pulse*.

"From the most remote antiquity physicians have been accustomed to judge of the relative states of health, and of disease, by the pulsation of the arteries, so termed from the notion, that they were tubes for the purpose of conveying air. The physicians of Greece used to examine the state of the pulse by applying the back of the hand to that part of the thorax where the pulsation of the heart is most discernible. By the Romans, however, it was known, that the pulse might be examined in various parts of the body."

"Only a slight sketch of the various indications to be derived from the pulse can be attempted on the present occasion. The brief tract of the late respectable Dr. Heberden affords an excellent example of strict philosophical disquisition on this subject. Bordieu's more extensive treatise, on the discovery of the crisis of diseases from the pulse, is chiefly translated from a still more voluminous work on the same subject by Dr. Solano, a Spanish physician, the study of which will amply repay the attention it demands. I have myself verified at the bed-side of a patient many of his minute, and what, perhaps, would be considered, by those who had not tried them by the test of experience, fanciful indications; and I have no doubt, that many more of his prognostics would be discovered to be founded in truth, by those who would carefully make the experiment.

"The Chinese physicians, it is well known, have long had the credit of paying very particular attention to the pulse: they even pretend to derive a much more minute and accurate knowledge of the state of the sick from that source than European practitioners lay any claim to. I possess a small volume, professing to be a view of the Chinese mode of judging of the pulse, translated by some of the original Jesuit missionaries. The patient is directed to be laid in bed, with his arm resting on a small cushion. The physician must be seated, and both parties are enjoined to remain calm, silent, and collected. The fingers are next to be applied in due succession, one after another, in order to judge of the compressibility of the artery. The Chinese do not infer solely from the rapidity of the pulsations. Their mode is to compare the number of pulsations of the artery with the intervals of the respiration of the patient. The number of pulsations of a man in moderate health they consider in relation to the time of a natural inspiration and expiration. Four beats of the pulse, during this period, they consider as indicating perfect health. If it exceeds five pulsations it is considered as too quick; if under that number, as too slow, respecting good health."

"The pulse may in general be considered as indicating the state of irritability of the living fibre."

"The pulse is full or small, in proportion to the quantity of blood circulating in the vessels; it is slow, quick, strong, or weak, in proportion to the vigour with which the heart contracts: hard or soft according to the tension and consequent resistance of the coats of the arteries. If the beating of the artery imparts a sensation similar to that of an elastic cord twitched under the fingers, whether slow or quick, the quantity of circulating blood may always be artificially diminished with safety and advantage.

"The organic pulse, not easily described in words, which Solano mentions, denotes the approaching crisis of disease, and is by him denominated the "lapis lydius Apollini." He also notices the pulsus myuris, or the creeping pulse, tapering away under the fingers like the tail of a rat, always an unfavourable symptom."

Dr. Buchan observes,

"In our inquiries concerning health, or the causes of disease, our attention should be particularly directed to ascertain the state of the various secretions, of the urine, fæces, saliva."

"Clear limpid urine, exceeding the healthy quantity, is a diagnostic symptom of that class of diseases usually denominated nervous.

"In order to enable a person to examine the urine with propriety, a portion of that passed first in the morning after sleep should be set apart in a cylindrical glass vessel. When this vessel is held between the eye and the light, if a cloudy appearance be observed suspended towards the top, it has been termed nubes, or nubecula; if in the middle, enæorema; if at the bottom, hypostasis.

"From the respective stations of these nubeculæ, much useful information was formerly supposed to be, and doubtless may be, derived. The hypostasis frequently indicates the crisis of fever, and a favourable termination of the disease. Small thread-like substances observed floating in the urine, are, in general, certain indications of recovery. Red particles, adhering strongly to the sides of the containing vessel, which patients commonly denominate a red sand, generally indicate disease of the liver. During a paroxysm of gout, the urine is commonly perfectly limpid, and deposits no sediment; but, with the decline of the fit, becomes extremely turbid, with copious deposits of floculent matter. Such appearance encourages a hope, that some remedy may yet be discovered for this painful disease, as it appears to be connected with the suppression of some matter, that ought to be secreted from the system.

"Quantity of urine greatly augmented, accompanied with sweet odour and taste, indicates diabetes."

"Very important information, especially respecting the diseases of children, is to be derived from the inspection of the alvine discharges. Notwithstanding the old sarcasm, that "Fæces et urina sunt medicis prandia prima," no false delicacy or misplaced pride should deter a man, determined to discharge his professional duties conscientiously, from ascertaining, by the evidence of his senses, the real appearance of the fæces." 55.

Dr. Buchan now retrogrades a little to notice the appearance of the *tongue*:

"The condition of the stomach and alimentary canal, or the chylopoetic viscera, as they have been termed, are in general inferred from the appearance of the tongue, the inspection of which should never be omitted in the examination of a patient. When the tongue appears parched without thirst in fever, or is affected with a tremulous motion, it is an unfavourable symptom. When red and tremulous, it betokens the approach of diarrhœa. When extremely red in inflammatory sore throat, or pleurisy, it is symptomatic of danger. A thin dark-coloured pellicle, extending along the centre of the tongue, indicates, I think, the presence of hydro-thorax."

"A foul, or loaded tongue, is commonly supposed to indicate the necessity of purgatives. In many instances, this is certainly the case. In that species of fever denominated gastric, or bilious, as it occurs in this country, the tongue is in general very much loaded; but under the action of frequent purging, I have observed this appearance to increase; and if the evacuant system be persisted in, the patient sinks, and death ensues. This fact particularly demands the attention of the younger practitioner." 58.

On *thirst*, and on the *appetite*, Dr. Buchan has the following notices:

"Thirst is symptomatic of indigestion, frequently a precursor of dropsy, although drinking freely of weak liquors is not, as is vulgarly thought, a cause, but a concomitant of that complaint. Deficient appetite accompanies, but does not cause fever. An unnatural craving for food is often the precursor of disease, especially of apoplexy.

"In chronic, and indeed, often in acute diseases, an eager craving for food is frequently the precursor of death: this fact is so familiar to the common people of the North, that it is commonly called the *yeard hunger*. The immediate approach of death is frequently indicated by a strong propensity to exonerate the bowels, and sick persons often expire during the effort. I think I have observed several instances of death being accelerated by the injudicious exhibition of an irritating cathartic, in cases of extreme debility." 60.

In this place, our author quits the subject of symptomatology, and treats of the state of medicine in Egypt—of the Pythagorean philosophy—of the doctrine of critical days, and the power of numbers—of the influence of the moon—of the diurnal motion of the earth, &c. He then skips back to his original topic, and observes,

"Care should be taken to distinguish the rigor, which precedes the attack of fever, from that by which the commencement of internal suppuration or deposition of pus is indicated. Two instances have come within my knowledge, in which a practitioner was so far deceived by an access of rigour, as to prescribe an arsenical preparation for the cure of ague, which, in one case was symptomatic of suppuration of the testicle, and the other of lumbar abscess." 78.

Of the appearances of the *eye*, Dr. Buchan remarks,

"A yellow tinge of the cornea indicates some derangement of the organs subservient to the secretion of the bile.

"A dark blue or cerulean appearance of the same part, is a pathognomonic symptom of diseased spleen. I discovered the certainty of this symptom in consequence of having an opportunity of seeing a number of men, who returned from the unfortunate expedition to Walcheren. In all of them this peculiar colour of the cornea was strikingly obvious. The bodies of almost all who died were examined, and in every case, the spleen was found diseased and enlarged, in some cases weighing not less than nine pounds.

"When the cornea appears of a pearly whiteness, accompanied with an expanded pupil, hollowness of the temples, the teeth generally sound, but with a bluish tinge, a tendency to phthisis pulmonalis may always be suspected.

"A peculiar bright sparkling appearance of the eyes, together with hurried and irregular motions of these organs, denote the approach of delirium or insanity. The presence of the latter disease is peculiarly indicated, according to the observations of Dr. Haslam, by a laxity of the integuments covering the occiput.

"A dull, prominent eye, betokens a propensity to apoplexy, as does also enlargement and fullness of the tongue. In advanced life, this complaint is frequently produced by venous plethora; in such cases, blood-letting is by no means always indicated as a remedy.

"Distended pupils indicate diminished irritability.

"Thickness of the eye-lids and alæ nasi are symptomatic of the scrofulous diathesis. When this fulness of the eye-lids is accompanied with redness on the margins, caused by inflammation of the ducts of the sebacious glands, it generally betokens a propensity to over-indulgence in fermented or distilled liquors."

"Even the particular kind of intoxicating liquor to which a person is addicted, may frequently be surmised from the physiognomy. Indulgence in vinous potations produces turgidity of the eyes, and a dark red inclining to purple hue of the complexion. Beer, a yellow bloated countenance, enlargement of the abdomen, and slowness of muscular action, well exemplified in the general condition of the draymen of London. Gin gives a leaden colour, deadness of the eye, emaciation, great depression of spirits, and diminution of muscular power. Brandy produces a peculiar ferocity of temper.

"I have been enabled to detect a secret attachment to the use of opium, a habit daily gaining ground in this country, from a peculiar, flaccid, greasy state of the skin, and a singular intolerance of light." 84.

Dr. Buchan again leaves the subject of symptomatology, and observes,

"It is the duty of the professors of medicine to investigate the nature of disease with a clear, unbiassed understanding, unprejudiced by theory, and influenced alone by a direct purpose to discover the truth. We ought carefully to be upon our guard against what Lord Bacon terms the *idolæ mentis*, the influence of which he considers as constituting one of the chief impediments to the advancement of human knowledge. An idola, or notion, may consist in referring every phenomenon to some preconceived theory, which has obtained possession of the understanding. Some practitioners, for example, suppose, that in this climate every anomalous form of disease may be referred to a scrofulous diathesis. Others, that all irregular constitutional complaints originate in a gouty disposition. When the nature of the venereal disease was not so well understood as at present, all undefined complaints were referred to some relics of that complaint lurking in the constitution. Boerhaave laid it down as a practical axiom, 'in dubio suspica luem.' Fifty years ago the diseases of persons of fashion were termed nervous; delicacy of the nervous system being at that period considered as indicating refinement of manners. In the days of Queen Elizabeth those slight irregularities of mind, which manifest their existence by oddity of manner, and complaints of unreal diseases, were attributed to deranged structure of the spleen." 93.

And further,

"It behoves medical men also to be upon their guard against wilful deception on the part of certain descriptions of patients. Such as attempts to conceal pregnancy, affectation of epileptic fits, and similar impostures." 97.

We cannot leave our author, without quoting the following feeling and amiable remarks :

"In the intercourse, which necessarily takes place between medica men and their fellow-creatures, when oppressed with sickness and with sorrow it may appear superfluous to recommend a due degree of at-l tention to humanity of conduct. Indeed, in many instances of human suffering, a considerable share of resolution is required to enable us to discharge our professional duty with propriety. Our firmness should, however, be always blended with gentleness.

"As early as the age of Hippocrates, the propriety of observing a due decorum in the conduct of the physician was well understood, as may be observed in the elegant treatise of that ancient author, concerning the vesture and demeanour becoming the profession. Galen acquaints us, that with a view to compose his mind into a proper frame for the discharge of his professional duties, he used to repeat to himself, morning and evening, the golden verses of Pythagoras. Temperance and prudence are virtues essential to the medical character.

"Neither in the records of ancient Greece nor Rome do we find mention made of any charitable institution for the relief of the poor or the sick. The stern republicans of Rome sent their unfortunate slaves, when afflicted with hopeless sickness, to perish upon a desert island in the Tyber.

"The first hospital for the sick, of which any account has been preserved, was established at Jerusalem by a devout Christian lady, named Paulina, for the reception and relief of such pilgrims as became afflicted with sickness during their visit to the holy sepulchre."

'I have frequently doubted whether medical men were justified in upbraiding persons, who seek relief from public charities, with those aberrations from the paths of morality, which may have reduced them to distress. The poor and afflicted are in general sufficiently aware of their own misconduct; and interrogatories frequently elicit peevish and unsatisfactory replies, even to our best intended queries. A physician is not strictly speaking the moral, but the medical reformer of his patients. The 'sacer est miser' should be a maxim ever present to the mind of the officer of a public charity. The 'vultus quasi miserantis,' a countenance as if commiserating the frailties of human nature, attributed to the fathers of Soloman's House by Lord Bacon, in his new Atlantis, aptly denotes what ought to be the deportment of a physician." 106.

We have not space or inclination to notice the tables of symptoms. The volume is so small altogether, that we advise our junior readers to possess themselves of it. But we cannot recommend it on stronger terms.

This article cannot be better concluded than by a statement of the following sentiment of the late celebrated and lamented Dr. Baillie, which he expressed to the writer of it, in the last interview which he had with that truly great man. Dr. Baillie observed, that the next real advancement in the science of physic would be made, when the symptoms of diseases should be studied with the same ardour and diligence with which the morbid anatomy had of late years been investigated. It is needless, after this, to multiply authorities; but we cannot resist the temptation of giving tbe following observation of the philosophic physician, Baglivi. "It is to be wished, I say, that some physicians, in all ages, had made it their business to pursue and improve the doctrine of signs: had that been done, questionless, the profession of physic had arrived at its acme long ere now, at least, as far as our mortal state will allow." *Book* II. *Chap*. viii.

